# How to be an integrated information theorist without losing your body

**DOI:** 10.3389/fncom.2024.1510066

**Published:** 2025-01-09

**Authors:** Ignacio Cea, Camilo Miguel Signorelli

**Affiliations:** ^1^Center for Research, Innovation and Creation, and Faculty of Religious Sciences and Philosophy, Temuco Catholic University, Temuco, Chile; ^2^Philosophy Department, Faculty of Philosophy and Humanities, Universidad Alberto Hurtado, Santiago, Chile; ^3^Department of Computer Science, University of Oxford, Oxford, United Kingdom; ^4^Center for Philosophy of Artificial Intelligence, Department of Communication, University of Copenhagen, Copenhagen, Denmark; ^5^Laboratory of Neurophysiology and Movement Biomechanics, Université Libre de Bruxelles, Brussels, Belgium

**Keywords:** Integrated Information Theory, consciousness science, computational neuroscience of consciousness, ontology of consciousness, formal metaphysics, scientific metaphysics, mathematics of consciousness, intrinsic ontology

## 1 Introduction

Integrated Information Theory 4.0 (IIT) is one of the leading frameworks in the neuroscience of consciousness (Consortium et al., 2023; Seth and Bayne, 2022; Signorelli et al., 2021). It aims to explain consciousness by mathematically formalizing its relation to cause-effect power and existence, while employing computational tools to investigate this experimentally (Zaeemzadeh and Tononi, 2024; Albantakis et al., 2023; Ellia et al., 2021). In principle, IIT can be used to assess both the level and content of consciousness in any physical system, such as the brain of a comatose patient or under anesthesia (Albantakis et al., 2023; Tononi et al., 2016).

More specifically, IIT conceives consciousness as an intrinsic structure of cause-effect powers, proposing that any conscious system exists for itself as a maximally unitary whole, irreducible to its parts (Albantakis et al., 2023; Ellia et al., 2021). This is mathematically formalized and computationally analyzed in terms of several measures of “integrated information.” In this article, we focus specifically on *maximal system integrated information* ($\varphi_{s}^{*}$). This is used by IIT to identify, among a set of candidate systems, the one(s) that supports consciousness, and hence “exists for itself” subjectively and irreducibly. In contrast, according to IIT's assumptions, systems that do not specify $\varphi_{s}^{*}$, at best, only “exist” from the perspective of another conscious entity, and hence do not “truly exist” (Albantakis et al., 2023; Koch, 2024; Tononi et al., 2022). In this way, IIT's measure of $\varphi_{s}^{*}$ is conceptually tied to both consciousness and an absolute, intrinsic form of existence, thus providing a computational neuroscience framework to quantitatively address questions related to both consciousness and ontology (i.e., about existence) that have been relegated to centuries of endless philosophical debates. At the same time, we acknowledge that, unlike familiar physical frameworks (like classical mechanics or thermodynamics) which can be introduced at progressive levels of mathematical detail and complexity (e.g., from *F* = ma to more advanced vectorial formulations), IIT's formalism remains comparatively opaque and harder to grasp.

Nevertheless, IIT still holds the potential to advance our understanding of questions related to ontology and consciousness through mathematical and computational means. But this potential is hindered by some key ontological assumptions of IIT, which lead to a problematic conceptual interpretation of its mathematical formalism, computational simulation results, and hypothetical scenarios allowed by the theory (Cea et al., 2024b,a, 2023). This underscores the crucial role that conceptual interpretation plays in scientific theories employing mathematical formalisms. History shows that reinterpreting pre-existing formalisms can be crucial for scientific advancement. For example, non-Euclidean geometry, developed by Gauss and later generalized by Riemann into higher-dimensional spaces, was conceptually reinterpreted by Einstein in his general relativity to describe the curvature of spacetime, addressing the limitations of Newtonian gravity, including its assumption of instantaneous information transfer in gravitational fields (Renn, 2007; Torretti, 1996). While our aims are far more modest and we are not comparing our work to Einstein's monumental achievements, reconsidering IIT's mathematical formalism from a new conceptual perspective might help address current limitations and enhance its explanatory power concerning the relationship between brain activity, consciousness, and ultimately, the concept of existence.

In the following, we first introduce the main principles of IIT (Section 2), focusing on the mathematical formalization of the theory's proposed marker of intrinsic, conscious existence (i.e., *maximal system integrated information* $\varphi_{s}^{*}$, and IIT's associated conceptual interpretation based on the ontological principles of (i) being, (ii) true existence, (iii) maximal existence, and (iv) “Great Divide of Being.” Next (Section 3), we briefly explain why these ontological assumptions are troublesome and motivate revision. Then (Section 4), we propose specific amendments to these ontological assumptions to improve the theory's overall theoretical robustness and thus, its capacity to address issues about consciousness and existence from a computational neuroscience perspective. We end with concluding remarks and propose directions for future research (Section 5).

## 2 Main principles of IIT

Grounded in the purportedly essential properties of experience (i.e., the “phenomenal axioms”), IIT proposes six “postulates of physical existence,” which, according to the theory, define the necessary and sufficient conditions for any physical substrate to instantiate consciousness. In line with IIT's *Principle of Being* (PB), which states that “to be is to have cause–effect power” (Albantakis et al., 2023, p. 11), these postulates are framed in terms of cause-effect power that must satisfy: (i) existence, (ii) intrinsicality, (iii) information, (iv) integration, (v) exclusion, and (vi) composition.

The theory then mathematically formalizes these causal-physical postulates and applies them to simulated neural networks (i.e., candidate substrates/systems).^1^ As anticipated, we will focus on *maximal system integrated information* $\varphi_{s}^{*}$, which is based on *system integrated information* φ_*s*_, a quantity that computes the cause-effect power of a system as an irreducible whole (Albantakis et al., 2023; Marshall et al., 2023).^2^

Mathematically, φ_*s*_ is computed as the minimum between a substrate's *integrated cause information (*φ_*c*_*)*, and *integrated effect information (*φ_*e*_*)*, according to the formalism (Albantakis et al., 2023, p. 17, Equations 19–21):


$$\begin{array}{l} \varphi_{s}\left(T_{e},T_{c},s,\theta \right)=\min\left\{\varphi_{c}\;\left(T_{c},s,\theta \right),\varphi_{e}\left(T_{e},s,\theta \right)\right\}\; \end{array}$$

While φ_*c*_ and φ_*e*_ are computed as follows:

*Integrated effect information (*φ_*e*_*):*


$$\varphi_{e}\left(T_{e},s,\theta \right)=p_{e}\left({s^{\prime }}_{e}|s\right)\;{\left|\log\left(\frac{p_{e}\left({s^{\prime }}_{e}|s\right)}{p_{e}^{\theta }\left({s^{\prime }}_{e}|s\right)}\right)\right|}_{+}$$

*Integrated cause information (*φ_*c*_*):*


$$\varphi_{c}\left(T_{c},s,\theta \right)=p_{c}^{\leftarrow }\left({s^{\prime }}_{c}|s\right)\;{\left|\log\left(\frac{p_{c}\left(s|{s^{\prime }}_{c}\right)}{p_{c}^{\theta }\left(s|{s^{\prime }}_{c}\right)}\right)\right|}_{+}$$

Both φ_*c*_ and φ_*e*_ quantify the difference that a partition θ of the system makes, with respect to the probability with which the entire unpartitioned system specifies its past/cause state $s_{c}^{\prime }$ (for φ_*c*_), and its future/effect state $s_{e}^{\prime }$ (for φ_*e*_), given its current state *s*. The greater the cause/effect probabilities $p_{e}\left(s_{e}^{\prime }|s\right)$ and $p_{c}^{\leftarrow }\left(s_{c}^{\prime }|s\right)$ specified by the unpartitioned system, and the greater the difference when partitioned (evaluated by the logarithmic terms), the greater the *integrated cause/effect information*, and hence, its overall φ_*s*__._^3^

Given that IIT endorses the *principle of maximal existence* (PME), which posits that “what exists is what exists the most” (Albantakis et al., 2023, p. 11), the *complex* (i.e., consciousness substrate) is identified as the subset of interconnected units within a universe *U*_*k*_ with *maximal* system integrated information ($\varphi_{s}^{*}$), as formalized below (Albantakis et al., 2023, p. 19, Equation 24):


$$\begin{array}{l} \varphi_{s}^{*}\left({\text{T}}_{e},{\text{T}}_{c},u_{k}\right)=\underset{S\subseteq U_{k}}{\max}\varphi_{s}\left({\text{T}}_{e},{\text{T}}_{c},s\right) \end{array}$$

where *U*_*k*_ represents the set of units available at iteration *k*, and *S* denotes a candidate subset. The subset achieving the maximum value, $\varphi_{s}^{*}$, is selected as the complex for that iteration, consistent with the PME. Iteratively, once a complex is identified, its units are removed from the universe *U*_*k*_, and the search continues within the remaining units to identify the next, non-overlapping, maximal subset. This process ensures that at the end of the iterative search, “overlapping substrates with lower φ_*s*_ are thus excluded from existence” (Albantakis et al., 2023, p. 18). Crucially, according to IIT's ontological interpretation, this “exclusion from existence” is literal: systems that don't specify *maximal* φ_*s*_ (i.e., $\varphi_{s}^{*}$) don't exist “for themselves” in the irrefutable, self-evidencing, experiential sense; and hence, according to IIT, do not truly exist. Thus, only maximal φ_*s*_ complexes truly exist, as they exist for themselves as subjective experiences (Tononi et al., 2022; Albantakis et al., 2023; Koch, 2024).^4^

Accordingly, IIT assumes what Cea et al. (2023) call IIT's *principle of true existence*, namely that “only phenomenal existence is true existence” (p. 4), as well as an *eliminative* stance that denies mind-independent existence to all physical entities that do not maximize φ_*s*_ and hence are non-conscious. This is closely related to IIT's “Great Divide of Being” (Koch, 2024; Tononi et al., 2022; Tononi, 2017), which is “the divide between what truly exists in an absolute sense, in and of itself—namely conscious, intrinsic entities—and what only exist in a relative sense, for something else” (Tononi et al., 2022, p. 8). This entails that familiar physical objects that do not instantiate $\varphi_{s}^{*}$, and hence are non-conscious, like “bodies and organs, tables and rocks. . . *do not truly exist*” (Tononi et al., 2022, p. 8, italics added).^5^ In other words, since our bodies presumably do not specify maximal φ_*s*_ (i.e., $\varphi_{s}^{*}$) and therefore do not qualify as additional substrates of consciousness besides the main complex in our brains, IIT's ontological interpretation implies that our own bodies do not truly exist. At best, they merely exist as objects for some consciousness observing them: “since my body is a superset of my true PSC (i.e., neural complex), it is excluded from it—relegated to the realm of entities that only exist relatively, for an observer” (Tononi et al., 2022, p. 8).

## 3 Problems with IIT's ontological assumptions

In previous works, we have examined the problematic implications of these radical assumptions in detail (Cea et al., 2024a,b,c, 2023; Signorelli et al., 2023). Here, we will briefly introduce them and direct the reader to that literature for further details. First, IIT's ontological commitments create an explanatory tension with both common neuroscientific practice and IIT's own declared goal of explaining consciousness in physical terms: why attempt to explain consciousness in neuroscientific terms if it is considered ontologically primitive, while, in contrast, any non-conscious physical entity is deemed mind-dependent?(Signorelli et al., 2023). Second, IIT's ontology seems to entail that truly existing, conscious systems, can (i) be eliminated from existence solely by altering external, non-existent entities; (ii) be engineered out of nothing; and (iii) either phylogenetically originate from nothing or have existed since the beginning of the universe (Cea et al., 2024b,a).

Now, IIT could *prima facie* address these issues by invoking an “ontological dust” (Tononi et al., 2022) ultimately composed of minimally conscious monads (indivisible units) (Hendren et al., 2024). However, there are several problems with the latter (Cea et al., 2024a,c). First, there are compelling reasons to think of a deep link between life and consciousness (Cea and Martínez-Pernía, 2023; Damasio and Damasio, 2024; Seth, 2024; Thompson, 2007), and thus against the idea of non-living, minimally conscious, indivisible particles of intrinsic existence. Second, the notion of monads seems to contradict IIT's own formalism (Cea et al., 2024c), which requires–to apply the integration postulate–that valid partitions of a system into at least two non-overlapping, non-empty parts, are possible (Albantakis et al., 2023). Otherwise, φ_*s*_ is not computable as such, but has to be replaced in practice by the measure of *intrinsic information*, which is insufficient for consciousness according to IIT's postulates (Cea et al., 2024c).

In sum, IIT's Great Divide of Being, along with its principles of true existence and eliminativism regarding non-conscious entities, raises several important issues.^6^ To address these concerns and align better with neuroscience practice, we propose minimal conceptual adjustments for IIT to adopt *causal-physical realism*, which asserts that non-$\varphi_{s}^{*}$, non-conscious systems may also truly exist if they have cause-effect power. This conceptual revision allows a reinterpretation of IIT's formalism such that maximal φ_*s*_ (i.e., $\varphi_{s}^{*}$) is no longer the exclusionary marker of a truly existent entity, but just the marker of consciousness, while causally powerful non-conscious entities may also be acknowledged to exist.

## 4 Proposed revisions to IIT's ontological assumptions

In the following, we propose that for IIT to endorse causal-physical realism and overcome the many problems we briefly sketched in the previous section, the theory should: (i) reject the principle of true existence (PTE; and associated Great Divide of Being) (Section 4.1), (ii) modify the principle of maximal existence (PME) (Section 4.2), and (iii) endorse a realistic, not merely operational, principle of being (PB) (Section 4.3).

### 4.1 Rejecting the principle of true existence and great divide of being

According to IIT's principle of true existence (hereafter “PTE,” Cea et al., 2023), only consciousness (i.e., phenomenal existence) is true existence, as it is “the only existence worth having—what we might call true existence” (Tononi et al., 2022, p. 8). While there is no systematic philosophical defense of PTE in the IIT literature, its core intuition seems to be that true, absolute existence is self-evident and immediately known by itself, a condition only conscious, intrinsically existing entities can meet: “consciousness truly exists because it exists for itself—it exists absolutely” (Tononi et al., 2022, p. 9). Therefore, only consciousness would truly exist, as only it exists for itself.

We have two worries about this intuition. First, it seems to rest on a *necessity-sufficiency equivocation*, conflating self-consciousness as a *sufficient* condition for the truth of one's existence (inspired by the Cartesian “cogito ergo sum”) with self-consciousness as a *necessary* condition for existence (as implied by PTE). One could argue that self-consciousness is sufficient to prove one's existence but reject the stronger claim that self-consciousness is required to exist. The Cartesian intuition supports the idea that “entities that exist for themselves truly exist” (a sufficiency claim), but this is compatible with the existence of non-conscious physical entities, not entailing that “*only* entities that exist for themselves truly exist” (a necessity claim).

Second, IIT's motivation to adopt PTE may also stem from an *epistemic-ontological equivocation*. While consciousness may entail *knowing* that one exists, there is no clear reason why this knowledge is inextricably tied to *existence*. However, IIT conflates this epistemic fact—self-*known* existence (“existing *for* itself”)—with the ontological fact of truly existing (“existing *in* itself”). We see no reason why something must *know* that it exists in order to *exist* (e.g. why a non-conscious stone cannot exist if it doesn't know its existence?). In short, “existing in itself” (true existence) does not imply “existing for itself” (self-aware existence), and thus the latter is not a necessary condition for the former.

In sum, we are happy to grant the Cartesian intuition according to which being conscious about one's existence may *suffice* for the truth of one's existence^7^ (a *sufficiency* claim) which IIT may safely embrace to assert the intrinsic existence of subjective experience based on its epistemic certainty and immediacy. This epistemic and phenomenological primacy of consciousness could position IIT in dialogue with the enactive approach and its neurophenomenological method, where first-person experience plays a foundational role in the scientific study of the mind (Varela, 1996; Varela et al., 2016; Signorelli et al., 2023). However, we see no reason to accept IIT's stronger thesis that in order to exist, an entity must “exist for itself (phenomenally)” (a *necessity* claim), which underlies the theory's principle of true existence and associated “Great Divide of Being.” Therefore, we propose that IIT theorists set aside this necessity claim and its associated theses to open up the conceptual possibility that non-conscious systems might exist in themselves, independently from any consciousness. In other words, without the problematic necessity claim underlying the PTE, the “Great Divide” between truly existing conscious entities and the merely relative, observer-dependent “existence” attributed to non-conscious entities disappears.

### 4.2 Revising the principle of maximal existence (PME)

The PME states that “what exists is what exists the most” (Albantakis et al., 2023, p. 11), meaning the system with maximal integrated information ($\varphi_{s}^{*}$) truly exists, while others are “excluded from existence” (Albantakis et al., 2023, p. 12). To allow for *causal-physical realism*, IIT should adopt our revised principle of maximal *conscious* existence (PMCE): “what exists *consciously* is what *holistically* (i.e., as a whole) exists the most.” Instead of determining, among overlapping systems, that the one with maximal φ_*s*_ is the only one conscious *and that truly exists*, the revised principle asserts that $\varphi_{s}^{*}$ only indicates consciousness, not exclusive existence. This aligns with IIT's method for identifying complexes, but without excluding non-$\varphi_{s}^{*}$ systems from existence. In other words, our proposed conceptual revision enables IIT to identify conscious systems as $\varphi_{s}^{*}$-specifying networks, and distinguish them from non-conscious ones (non-$\varphi_{s}^{*}$-specifying), without further claiming that the latter do not truly exist. This way, claims about consciousness based on measuring $\varphi_{s}^{*}$ are dissociated from claims about genuine existence, allowing the latter notion to apply to a broader class of systems, including non-conscious ones. However, we also seek to avoid the unrestrained proliferation of entities. This is where our *revised principle of being* becomes central.

### 4.3 Revising the principle of being

IIT's principle of being (“PB”) asserts that “in physical, operational terms, to exist requires being able to take and make a difference” (Albantakis et al., 2023, p. 11). In other words, operational, physical existence is causal power. The qualifier “operational” is very important. PB is understood “in terms of what can be observed and manipulated” (Albantakis et al., 2023, p. 2) by intrinsic entities such as conscious neuroscientists. But it does not guarantee “true existence,” which only conscious intrinsic entities enjoy (Albantakis et al., 2023; Tononi et al., 2022; Koch, 2024).

However, we suggest IIT to adopt a fully realistic version of the PB, what we may call the *principle of realistic being* (PRB).^8^ According to it, *to truly exist is to have causal power*. As with all previous suggested theoretical modifications, endorsing this revised *principle of realistic being* does not entail any changes to IIT's current methodology and mathematical formalism. But *conceptually*, it allows the theory to endorse a full-blown realism about all non-conscious (non-$\varphi_{s}^{*}$ specifying)–but causally powerful–physical entities, and thus, potentially resolve all the issues we briefly outlined, which stem from non-conscious systems with causal power being excluded from true existence. In the technical terms of IIT, we propose that specifying both non-maximal φ_*s*_, and even just *intrinsic information* (cause-effect power), may be sufficient for an entity to exist genuinely, even if not consciously.

## 5 Concluding remarks

In sum, our analysis suggests that IIT needs to endorse *causal-physical realism*, and to achieve that, (i) reject the *principle of true existence* (PTE) (and associated Great Divide of Being); (ii) endorse the revised *principle of maximal conscious existence*: “what exists *consciously* is what *holistically* exists the most” (PMCE); and (iii) endorse the revised *principle of realistic being* (“PRB”), according to which “to truly exist is to have causal power.” This would allow IIT theorists to pursue their current neuroscientific methodology and computational framework to find the physical substrate of consciousness (i.e., complex) and unfold its cause-effect structure, without conceptually entailing the rejection of the mind-independent, genuine existence of the non-conscious parts of their own brains and bodies.

Future theoretical research should assess the types of entities allowed by IIT's formalism, once the “Great Divide of Being” is overcome. For instance, what is the ontological difference between entities that only specify positive values of *intrinsic information* but zero *system integrated information*, compared to entities that do specify positive values of the latter? Presumably, both exist in virtue of having cause-effect power, but only the latter present *causal emergence* (Hoel et al., 2013; Mediano et al., 2022; Hoel et al., 2016).

Additionally, future research could explore IIT's potential to integrate theoretical insights and empirical findings from embodied approaches, which propose that the non-neural body, far from “existing” solely from the perspective of the conscious brain, is structurally and dynamically intertwined with it (Thompson and Cosmelli, 2012; Thompson and Varela, 2001) and fundamental to understanding both the origins of our mathematical capacities (Lakoff and Nunez, 2000) and the very feeling of existence at the root of all consciousness (Thompson and Cosmelli, 2012; Seth, 2021, 2024; Damasio and Damasio, 2023, 2024; Thompson and Varela, 2001; Cea and Martínez-Pernía, 2023; Ratcliffe, 2020).

## Footnotes

^1^In IIT, the causal state transitions of a system are captured by its transition probability matrix (TPM), constructed by perturbing the system into all possible states and observing the resulting states. This is similar to a Markov chain, in that both describe state transitions probabilistically based on current states, but also differ because IIT's TPMs marginalize external influences to focus on the internal causal relationships of a system, and encode interventional, rather than purely observational probabilities. Thus, transitions between conscious states in a substrate are operationally tracked by its TPM and the corresponding unfolded cause-effect structure (= conscious state) at each time step. However, according to IIT's ontological narrative, what truly happens is that subjective conscious states successively cause one another irreducibly, as only consciousness “truly exists” (Tononi et al., 2022). This creates a problematic tension between IIT's operational framework and its ontological claims concerning the causality of consciousness, a point critiqued in other work (Signorelli et al., 2023). Many thanks to reviewer 1 for pressing these and other relevant points addressed below.

^2^In the IIT 4.0 formalism, there are other important measures like the *integrated information for distinctions* ϕ_*d*_*, integrated information for relations* ϕ_*r*_*, structure integrated information* Φ, and the Φ*-structure*. However, given the scope of this article, we focus on φ_*s*_ and $\varphi_{s}^{*}$. We refer the reader to Albantakis et al. (2023) for further details.

^3^IIT computes φ_*s*_ using the Minimum Information Partition (MIP), which is the partition that minimizes integrated information (Albantakis et al., 2023; Marshall et al., 2023).

^4^In contrast to common computational approaches that prioritize input-output functions as critical for understanding conscious processes, IIT targets the internal causal structure of a system as explanatorily central i.e., how its constitutive mechanisms affect each other and the dynamical evolution of the whole system. Thus, while a complex does have clear boundaries defined by the subset of interacting units that maximize the value of φ_*s*_ compared to overlapping subsets, and it can receive/send input/output signals from/to units outside its boundaries, these external interactions are not constitutive of the system's intrinsic cause-effect power and consciousness. Nonetheless, a system's internal causal structure should somehow match the causal structure of the environment in perceptual experience, otherwise both individual and shared perception of the external world among different people would be impossible. This is currently an important limitation of IIT, but efforts to address it are ongoing (Mayner et al., 2024). Additionally, IIT provides, in principle, a framework to measure the internal causal structure of systems computationally through interventions and state-transition analyses. However, practical limitations, particularly in applying these methods to biological systems like the human brain, remain significant. We thank Reviewer 1 for pressing these important points.

^5^From IIT's perspective, only φ_*s*_-maximal systems are conscious entities that truly exist as *subjects* experiencing their own existence and, potentially, an external world (Tononi et al., 2022; Cea et al., 2023). For instance, a φ_*s*_-maximal brain region within a conscious neuroscientist would constitute a genuine subject. In turn, whether a patient observed by the neuroscientist is another intrinsically existing subject or merely an object within the neuroscientist's experience depends on whether the patient also possesses a φ_*s*_-maximal brain region.

^6^Another important issue is the ontological relationship, in IIT, between a physical substrate and its consciousness. In previous work we argued that a physical substrate ontologically reduces to its intrinsic Φ*-*structure, which in turn ontologically reduces to its inner subjective experience. In other words, what truly exists would be the subjective experience, but it could be observed from an extrinsic point of view as a physical substrate, whose experience can be described–in scientific-theoretical terms–as a Φ*-*structure (Cea et al., 2023). Thus, although subjective experience is ontologically primary and not directly observable from the third-person perspective, IIT suggests that it can be scientifically represented by the corresponding Φ-structure, which, in principle, can be computed for any physical system modeled as a causal stochastic network, according to Equations 57, 58 in Albantakis et al. (2023, p. 29). However, this is not yet feasible for realistic systems, due to combinatorial explosions in the calculations, manipulations and observations needed, making it currently practical only for idealized systems with a few units. This is a significant limitation that requires further development (e.g. Zaeemzadeh and Tononi, 2024) to enable rigorous empirical testing of IIT. Without this, IIT remains only “testable in principle”—a shortcoming for a theory that seeks to scientifically explain consciousness. Nevertheless, practical tools like the Perturbational Complexity Index (PCI), inspired by IIT, have provided some initial empirical validation for the theory by reliably estimating consciousness levels in clinical settings (Massimini et al., 2009; Casarotto et al., 2024). We thank again reviewer 1 for highlighting this issue.

^7^To clarify, this doesn't necessarily entail the truth of the existence of one's ego, soul or substantial self, but only the existence of one's consciousness.

^8^In contrast to IIT's current principle of being, which, for the sake of conceptual precision, may be better called the *principle of operational being*. Notice also that our revised “principle of realistic being” assumes full realism about causal powers, while IIT's current PB seems committed to a mere operational/instrumentalist view of causal powers.

## References

[B1] Albantakis L. Barbosa L. Findlay G. Grasso M. Haun A. M. Marshall W.. (2023). Integrated Information Theory (IIT) 4.0: formulating the properties of phenomenal existence in physical terms. PLOS Comput. Biol. 19:e1011465. 10.1371/journal.pcbi.1011465 37847724 PMC10581496

[B2] Casarotto S. Hassan G. Rosanova M. Sarasso S. Derchi C.-C. Trimarchi P. D.. (2024). Dissociations between spontaneous electroencephalographic features and the perturbational complexity index in the minimally conscious state. Eur. J. Neurosci. 59, 934–947. 10.1111/ejn.16299 38440949

[B3] Cea I. Martínez-Pernía D. (2023). Continuous organismic sentience as the integration of core affect and vitality. J. Conscious. Stud. 30, 7–33. 10.53765/20512201.30.3.007

[B4] Cea I. Negro N. Signorelli C. M. (2023). The fundamental tension in integrated information theory 4.0's realist idealism. Entropy 25:1453. 10.3390/e25101453 37895574 PMC10606349

[B5] Cea I. Negro N. Signorelli C. M. (2024a). Big bang consciousness: IIT 4.0 and the origin of subjective experience. PsyArXiv. 10.31234/osf.io/q9zmr

[B6] Cea I. Negro N. Signorelli C. M. (2024b). Only consciousness truly exists? Two problems for IIT 4.0's ontology. Front. Psychol. 15:1485433. 10.3389/fpsyg.2024.1485433 39507083 PMC11537854

[B7] Cea I. Negro N. Signorelli C. M. (2024c). Why Phi-Monads Cannot Exist: IIT 4.0 and the Formal Impossibility of Indivisible Units of Consciousness.

[B8] Consortium C. Ferrante O. Gorska-Klimowska U. Henin S. Hirschhorn R. Khalaf A.. (2023). An adversarial collaboration to critically evaluate theories of consciousness. bioRxiv 2023–26. 10.1101/2023.06.23.546249

[B9] Damasio A. Damasio H. (2023). Feelings are the source of consciousness. Neural Comput. 35, 277–286. 10.1162/neco_a_01521 35896152

[B10] Damasio A. Damasio H. (2024). Homeostatic feelings and the emergence of consciousness. J. Cogn. Neurosc. 36, 1653–1659. 10.1162/jocn_a_02119 38319678

[B11] Ellia F. Hendren J. Grasso M. Kozma C. Mindt G. Lang J. P.. (2021). Consciousness and the fallacy of misplaced objectivity. Neurosci. Conscious. 2021, 1–12. 10.1093/nc/niab032 34667639 PMC8519344

[B12] Hendren J. Grasso M. Juel B. E. Tononi G. (2024). ‘*Glossary of IIT Terms.' IIT Wiki. Center for sleep and consciousness UW–Madison*. Available at: https://www.iit.wiki/glossary#h.otx4utf9u8sn

[B13] Hoel E. P Albantakis L. Marshall W. Tononi G. (2016). Can the macro beat the micro? Integrated information across spatiotemporal scales. Neurosci. Conscious. 2016:niw012. 10.1093/nc/niw012 30788150 PMC6367968

[B14] Hoel E. P. Albantakis L. Tononi G. (2013). Quantifying causal emergence shows that macro can beat micro. Proc. Natl. Acad. Sci. 110, 19790–19795. 10.1073/pnas.1314922110 24248356 PMC3856819

[B15] Koch C. (2024). Then I Am Myself the World: *What Consciousness Is and How to Expand It*. New York, NY Basic Books.

[B16] Lakoff G. Nunez R. (2000). Where Mathematics Comes From. New York, NY: Basic Books.

[B17] Marshall W. Grasso M. Mayner W. G. P. Zaeemzadeh A. Barbosa L. S. Chastain E.. (2023). System Integrated Information. Entropy 25:334. 10.3390/e25020334 36832700 PMC9955253

[B18] Massimini M. Boly M. Casali A. Rosanova M. Tononi G. (2009). A perturbational approach for evaluating the brain's capacity for consciousness. Prog. Brain Res. 177, 201–214. 10.1016/S0079-6123(09)17714-2 19818903

[B19] Mayner W. G. P. Juel B. E. Tononi G. (2024). Meaning, perception, and matching: quantifying how the structure of experience matches the environment.

[B20] Mediano P. A. M. Rosas F. E. Luppi A. I. Jensen H. J. Seth A. K. Barrett A. B.. (2022). Greater than the parts: a review of the information decomposition approach to causal emergence. Philos. Trans. R. Soc. A 380:20210246. 10.1098/rsta.2021.0246 35599558 PMC9125226

[B21] Ratcliffe M. (2020). “Existential feelings,” in The Routledge Handbook of Phenomenology of Emotion, eds. T. Szanto, and H. Landweer (London: Routledge), 620. 10.4324/9781315180786-25

[B22] Renn J. (2007). The Genesis of General Relativity: Sources and Interpretations, Vol. 250. Cham: Springer Science and Business Media.

[B23] Seth A. (2021). Being You: A New Science of Consciousness. New York, NY: Penguin.

[B24] Seth A. (2024). Conscious artificial intelligence and biological naturalism. PsyArXiv. 10.31234/osf.io/tz6an35317021

[B25] Seth A. K. Bayne T. (2022). Theories of consciousness. Nat. Rev. Neurosci. 23, 439–452. 10.1038/s41583-022-00587-4 35505255

[B26] Signorelli C. M. Cea I. Prentner R. (2023). We need to explain subjective experience, but its explanation may not be mechanistic. PsyArXiv. 10.31234/osf.io/e6kdg PMC13156949 42110565

[B27] Signorelli C. M. Szczotka J. Prentner R. (2021). Explanatory profiles of models of consciousness-towards a systematic classification. Neurosci. Conscious. 2021:niab021. 10.1093/nc/niab021 34457353 PMC8396118

[B28] Thompson E. (2007). Mind in Life: Biology, Phenomenology, and the Sciences of Mind. Cambridge, MA: Harvard University Press.

[B29] Thompson E. Cosmelli D. (2012). Brain in a vat or body in a world? brainbound versus enactive views of experience. Philos. Topics 39, 163–80. 10.5840/philtopics20113911937091293

[B30] Thompson E. Varela F. (2001). Radical embodiment: neural dynamics and consciousness. Trends Cogn. Sci. 5, 418–425. 10.1016/S1364-6613(00)01750-2 11707380

[B31] Tononi G. (2017). “Integrated information theory of consciousness: some ontological considerations,” in The Blackwell Companion to Consciousness, 2nd Edn, eds. S. Schneider, and M. Velmans (West Sussex: Wiley Online Library), 621–633. 10.1002/9781119132363.ch44

[B32] Tononi G. Albantakis L. Boly M. Cirelli C. Koch C. (2022). Only what exists can cause: an intrinsic view of free will. arXiv [Preprint]. arXiv:2206.02069. 10.48550/arXiv.2206.0206914577287

[B33] Tononi G. Boly M. Massimini M. Koch C. (2016). Integrated information theory: from consciousness to its physical substrate. Nat. Rev. Neurosci. 17, 450–461. 10.1038/nrn.2016.44 27225071

[B34] Torretti R. (1996). Relativity and Geometry. North Chelmsford, MA: Courier Corporation.

[B35] Varela F. (1996). Neurophenomenology: a methodological remedy for the hard problem. J. Conscious. Stud. 3, 330–349.

[B36] Varela F. Thompson E. Rosch E. (2016). *T*he Embodied Mind*: Cognitive Science and Human Experience. Revised Ed*. Cambridge, MA: The MIT Press. 10.7551/mitpress/9780262529365.001.0001

[B37] Zaeemzadeh A. Tononi C. (2024). Upper bounds for integrated information. PLOS Comput. Biol. 20:e1012323. 10.1371/journal.pcbi.1012323 39102449 PMC11326638

